# Clinical and echocardiographic characteristics of adults with atrial septal aneurysm

**DOI:** 10.47487/apcyccv.v6i4.541

**Published:** 2025-12-29

**Authors:** Alfonso L. León-Burgos

**Affiliations:** 1 Hospital de Alta Complejidad Virgen de la Puerta, Trujillo, Perú. Hospital de Alta Complejidad Virgen de la Puerta Trujillo Perú

**Keywords:** Aneurysm, Heart Septal Defects, Atrial, Echocardiography, Aneurisma, Malformaciones del Tabique Interatrial, Ecocardiografía

## Abstract

**Objective.:**

To determine the prevalence and describe the clinical and echocardiographic characteristics of adults with atrial septal aneurysm (ASA).

**Materials and methods.:**

A cross-sectional, descriptive study was conducted. The population consisted of patients diagnosed with ASA by transthoracic echocardiography.

**Results.:**

The prevalence was 2%. The median age was 71 years, and females predominated (58.8%). ASA with right bulging represented 74.8%. The most frequent comorbidity was hypertension (64.1%). Atrial tachyarrhythmias were the most prevalent arrhythmias, and the most common valvular disease was mitral regurgitation (26.7%).

**Conclusions.:**

The prevalence of ASA was similar to that reported in the literature; females predominated, the most prevalent comorbidity was hypertension, atrial tachyarrhythmias were the most frequent arrhythmias, ASA with right bulging was the most common, and mitral regurgitation was the most prevalent valvulopathy.

## RESUMEN

**Objetivo.:**

Determinar la prevalencia, describir las características clínicas y ecocardiográficas de adultos con aneurisma del *septum* interauricular (ASIA). **Materiales y métodos.** Estudio transversal, descriptivo cuya población estuvo conformada por pacientes con ASIA diagnosticados por ecocardiografía transtorácica.

**Resultados.:**

La prevalencia fue del 2%, la edad tuvo una mediana de 71 años, el sexo femenino predominó con 58,8%. Los ASIA con desviación a la derecha representaron el 74,8%. La comorbilidad más frecuente fue la HTA con 64,1%, las taquiarritmias auriculares fueron las arritmias más prevalentes y la valvulopatía más frecuente fue la insuficiencia mitral con 26,7%.

**Conclusiones.:**

La prevalencia del ASIA fue similar a la reportada en la literatura, predominó el sexo femenino, la comorbilidad más prevalente fue la HTA, las taquiarritmias auriculares fueron las arritmias más frecuentes, los ASIA con desviación a la derecha fueron los más comunes y la insuficiencia mitral fue la valvulopatía más prevalente.

## Introduction

Atrial septal aneurysm (ASA) is a redundancy or saccular deformity of the interatrial septum. It is defined as an excursion of the septum toward one atrium exceeding 10 mm relative to the plane of the septum ^1^, with a base width greater than 15 mm ^2^, or alternatively as a combined total displacement toward both atria of at least 15 mm ^1^.

ASA diagnosed by transthoracic echocardiography (TTE) has a reported prevalence in adults ranging from 0.2% ^3^ to 2.4% ^4^. Its clinical significance remains incompletely defined; however, multiple studies have reported associations with cardioembolic stroke ^5^^,^^6^, mitral valve prolapse (MVP) ^7^, patent foramen ovale (PFO) ^8^, atrial septal defect (ASD) ^9^, aortic regurgitation, mitral regurgitation (MR) (4), cardiac arrhythmias ^4^^,^^10^, and migraine ^11^.

To date, no studies have been conducted in our setting to assess the prevalence of ASA or the conditions associated with this entity. Given the clinical relevance of obtaining this information, the present study was undertaken to determine the clinical and echocardiographic characteristics of adult patients with this diagnosis.

## Materials and methods

### Study design

A cross-sectional, descriptive study was conducted at High-Complexity Hospital Virgen de la Puerta (Trujillo, Peru) between 2015 and 2019, and in 2022.

### Study population

Patients aged 18 years or older with a diagnosis of ASA established by TTE were included. We excluded patients with poor acoustic windows, a history of cardiac surgery involving the interatrial septum, percutaneous closure of a PFO or ASD, percutaneous balloon mitral commissurotomy, severe mitral stenosis, or pulmonary hypertension.

### Procedures

Echocardiographic examinations were performed using a Philips HD 11Xe system equipped with a 3-MHz cardiac transducer.

### Variables

Demographic and clinical data, associated comorbidities, and echocardiographic variables were collected. 

### Operational definition of atrial septal aneurysm

Excursion of the interatrial septum, typically at the level of the fossa ovalis, toward one or both atria fulfilling at least one of the following criteria: displacement of the septum from the interatrial plane toward one atrium greater than 10 mm, or a combined total displacement toward both atria of 15 mm ^1^, with a minimum base width of 15 mm at the aneurysmal base in both cases ^2^.

ASA was classified according to the proposal by Olivares-Reyes *et al.*^2^ as follows: type 1R, protrusion toward the right atrium; type 2L, protrusion toward the left atrium; type 3RL, predominant protrusion toward the right atrium with a lesser excursion toward the left atrium; type 4LR, predominant protrusion toward the left atrium with a lesser excursion toward the right atrium; and type 5, when aneurysmal motion is bidirectional and equidistant.

### Statistical analysis

Quantitative variables were summarised using the mean and standard deviation or the median and interquartile range, as appropriate, according to their distribution. Categorical variables were described using absolute frequencies and percentages. All data were analysed using SPSS statistical software, version 26.

### Ethical aspects

The study received approval from the Research and Ethics Committee of the La Libertad Healthcare Network. All information obtained during the investigation was handled in accordance with established research standards, as outlined in the Declaration of Helsinki.

## Results

During the 2015-2019 period and in 2022, a total of 6,391 TTE examinations eligible for the study were performed. Of these, 131 patients were diagnosed with ASA, yielding a prevalence of 2.0%. The median age was 71 years, with the 60-79-year age group being the most frequent, accounting for 62.6% of cases (Table 1). Female sex predominated overall (58.8%). When analysed by age group, female predominance was observed across all groups except among individuals aged 80 years or older, in whom male sex was more frequent, representing 56.5% of cases (Table 2).

**Table 1:** Demographic, clinical, and echocardiographic characteristics of adult patients with atrial septal aneurysm.

Characteristics	Observed values
Demographic	
Age (years) ¥	71 (63-78)
<40, n (%)	6 (4.5)
40-80, n (%)	102 (77.9)
>80, n (%)	23 (17.6)
Male sex, n (%)	54 (41.2)
Clinical	
Weight (kg) ¥	62 (54.5-72.5)
Height (m) *	1.55 ± 0.09
BMI ¥	25.8 (23.0-28.7)
Comorbidities	
Hypertension, n (%)	44 (64.1)
Ischaemic stroke, n (%)	16 (12.2)
Atrial fibrillation, n (%)	14 (10.7)
Supraventricular extrasystoles, n (%)	4 (3.1)
Paroxysmal supraventricular tachycardia, n (%)	9 (6.9)
Atrial tachyarrhythmias, n (%)	24 (18.3)
Ventricular extrasystoles, n (%)	4 (3.1)
Ventricular tachycardia, n (%)	1 (0.8)
Heart failure, n (%)	8 (6.1)
Ischaemic heart disease, n (%)	14 (10.7)
Hypertrophic cardiomyopathy, n (%)	4 (3.1)
Congenital heart disease, n (%)	2 (1.5)
Aortic aneurysm, n (%)	2 (1.5)
Diabetes mellitus, n (%)	25 (19.1)
Neoplasia, n (%)	23 (17.6)
Hypothyroidism, n (%)	16 (12.2)
End-stage chronic kidney disease, n (%)	2 (1.5)
Diffuse interstitial lung disease, n (%)	2 (1.5)
Pulmonary fibrosis, n (%)	3 (2.3)
Chronic obstructive pulmonary disease, n (%)	1 (0.8)
Echocardiographic data	
Aneurysm type	
1R, n (%)	69 (52.6)
2L, n (%)	28 (21.4)
3RL, n (%)	29 (22.1)
4LR, n (%)	4 (3.1)
5, n (%)	1 (0.8)
Left ventricular ejection fraction ¥	59 (56-63)
<40%, n (%)	2 (1.5)
40-50%, n (%)	9 (6.9)
>50, n (%)	120 (91.6)
Left atrial area (cm²) ¥	21 (18-25)
Indexed left atrial volume (mL/m²) ¥	41.5 (33.5-53.8)
Patent foramen ovale, n (%)	2 (1.5)
Valvular heart disease	
Mitral regurgitation, n (%)	35 (26.7)
Aortic regurgitation, n (%)	31 (23.7)
Aortic stenosis, n (%)	2 (1.5)
Mitral stenosis, n (%)	1 (0.8)
Tricuspid regurgitation, n (%)	7 (5.3)
Pulmonary regurgitation, n (%)	3 (2.3)
E-wave velocity (cm/s) ¥	65 (54-85)
E/A ratio ¥	0.8 (0.6-1.2)
E/e' ratio ¥	9.4 (7.7-13.1)
Pulmonary artery systolic pressure (mmHg) ¥	28 (24-33)

BMI: body mass index

*: mean (standard deviation)

¥: median (interquartile range)

**Table 2:** Distribution of adult patients with atrial septal aneurysm according to age group.

Age group	Total	Male sex	IAS deviation to the right	IAS deviation to the left	Ischaemic stroke	Atrial fibrillation	Hypertension	Diabetes mellitus	Neoplasia	Mitral regurgitation	Aortic regurgitation
<40, n (%)	6 (4.6)	2 (33.3)	6 (100.0)	0 (0)	1 (16.7)	1 (16.7)	1 (16.7)	0 (0)	0 (0)	0 (0)	0 (0)
40-59, n (%)	18 (13.7)	6 (33.3)	16 (88.9)	2 (11.1)	0 (0)	1 (5.6)	8 (44.4)	5 (27.8)	1 (5.6)	4 (22.2)	0 (0)
60-79, n (%)	82 (62.6)	32 (39.0)	61 (74.4)	21 (25.6)	11 (13.4)	9 (10.9)	57 (69.5)	19 (23.2)	13 (15.9)	23 (28.0)	19 (23.2)
≥80, n (%)	25 (19.1)	14 (56.0)	15 (60.0)	10 (40.0)	4 (16.0)	3 (12.0)	18 (72.0)	1 (4.0)	9 (36.0)	8 (32.0)	12 (48.0)

IAS: interatrial septum

Rightward-deviating ASAs were the most common, accounting for 74.8% of cases (Table 1), and showed a decreasing trend in frequency with increasing age group (Table 2).

The most frequent comorbidity was hypertension, with a prevalence of 64.1%. Similarly, the prevalence of atrial fibrillation, ischaemic stroke, and neoplasms increased progressively with advancing age (Table 2). 

Among arrhythmias, atrial tachyarrhythmias were the most common, with atrial fibrillation present in 10.7% of cases.

Regarding congenital heart disease, two patients had a patent foramen ovale, one case of atrial septal defect with left-to-right shunt associated with a type 3RL ASA was identified, and one patient had a patent ductus arteriosus.

The most frequent valvular disorders were mitral regurgitation (26.7%) and aortic regurgitation (23.7%), considering mild, moderate, and severe grades. The prevalence of both valvular conditions increased with advancing age.

When patients were grouped according to the predominant direction of ASA deviation, those with leftward septal deviation tended to be older. In contrast, in the group with predominant rightward deviation, atrial tachyarrhythmias were more frequent; moreover, the indexed left atrial volume and the E-wave velocity were higher (Table 3).

**Table 3:** Demographic, clinical, and echocardiographic characteristics of adult patients according to the predominant direction of atrial septal aneurysm deviation.

Characteristics	Rightward deviation (n = 98)	Leftward deviation (n = 32)
Demographic		
Age (years) ¥	71 (62-77.8)	74.5 (67.8-81.3)
Male sex, n (%)	37 (37.8)	17 (53.1)
Clinical		
Weight (kg) ¥	61.5 (54-72)	66 (55.8-74.3)
Height (m) *	1.54 (0.1)	1.57 (0.1)
Body mass index ¥	25.7 (23-28.7)	27.3 (23.8-28.7)
Comorbidities		
Hypertension, n (%)	60 (61.2)	23 (71.8)
Ischaemic stroke, n (%)	15 (15.3)	1 (3.1)
Atrial fibrillation, n (%)	13 (13,3)	1 (3.1)
Supraventricular extrasystoles, n (%)	4 (4.1)	0 (0)
Paroxysmal supraventricular tachycardia, n (%)	8 (8.2)	1 (3.1)
Atrial tachyarrhythmias, n (%)	22 (22.4)	2 (6.3)
Ventricular extrasystoles, n (%)	4 (4.1)	0 (0)
Ventricular tachycardia, n (%)	1 (1.0)	0 (0)
Heart failure, n (%)	8 (8.2)	0 (0)
Ischaemic heart disease, n (%)	9 (9.2)	5 (15.6)
Hypertrophic cardiomyopathy, n (%)	3 (6.1)	1 (3.1)
Congenital heart disease, n (%)	2 (2.0)	0 (0)
Patent foramen ovale, n (%)	1 (1.0)	1 (3.1)
Aortic aneurysm, n (%)	0 (0)	2 (6.3)
Diabetes mellitus, n (%)	22 (22.5)	3 (9.4)
End-stage chronic kidney disease, n (%)	2 (2.0)	0 (0)
Diffuse interstitial lung disease, n (%)	1 (1.0)	1 (3.1)
Pulmonary fibrosis, n (%)	3 (3.1)	0 (0)
Chronic obstructive pulmonary disease, n (%)	0 (0)	1 (3.1)
Neoplasia, n (%)	16 (16.3)	7 (21.9)
Hypothyroidism, n (%)	12 (12.2)	3 (9.4)
Echocardiographic		
Left ventricular ejection fraction ¥	60 (56- 63)	59 (57.8-61.5)
Left atrial area (cm²) ¥	21.5 (18-25)	20 (18-24)
Indexed left atrial volume (mL/m²) ¥	42 (33.9-55.9)	35.4 (30.4-44.8)
E-wave velocity (cm/s) ¥	66.5 (57.5-92.8)	61.5 (42.8-68.5)
E/e' ratio ¥	10 (7.6-14.1)	8.7 (7.7-10.6)
Pulmonary artery systolic pressure (mmHg) ¥	28 (24-33)	26 (23-30.3)
Mitral regurgitation, n (%)	26 (26.5)	9 (28.1)
Aortic regurgitation, n (%)	24 (24.5)	7 (21.8)
Aortic stenosis, n (%)	1 (1.0)	1 (3.1)
Mitral stenosis, n (%)	1 (1.0)	0 (0)
Tricuspid regurgitation, n (%)	5 (5.1)	2 (6.2)
Pulmonary regurgitation, n (%)	1 (1.0)	2 (6.2)

¥: median (interquartile range).

*: mean (standard deviation).

**: Type 5 atrial septal aneurysm was excluded because it does not have a predominant direction of deviation.

## Discussion

In this study, the prevalence of ASA was 2.0%, a value that falls within the range reported in the literature, which varies from 1.2% to 4.9% across different population-based studies ^3^^,^^4^.

With respect to sex, a female predominance was observed, a finding consistent with that reported in several case series ^3^ and in larger registries, where the proportion of women ranges from 54% to 68% ^2^^,^^4^^,^^10^^,^^12^^-^^14^.

When patients were stratified by age group, this female predominance was evident only among those younger than 80 years. Concordantly, comparisons of age and the proportion of women with ASA in previously published studies ^2^^,^^4^^,^^10^^,^^12^^,^^13^^)^ also show a progressive decline in female predominance with increasing age, although the reasons underlying this trend remain unclear.

The patients’ age in the present study was higher than that reported in previous investigations ^2^^-^^4^^,^^10^^,^^12^^,^^13^^,^^15^. An older age among patients with ASA, as well as an increase in its frequency after the age of 50 years, has been described, suggesting a possible role of age in the pathophysiology of interatrial septal abnormalities, including ASA ^14^.

Regarding comorbidities, hypertension was the most frequent, in agreement with other studies ^2^^,^^4^^,^^10^^,^^12^^,^^14^, although with a higher prevalence than that observed in most of them ^4^^,^^10^^,^^12^^,^^13^. This difference may be explained, at least in part, by the older age of patients in our sample, given that the prevalence of hypertension increases progressively with age ^16^.

Ischaemic stroke was present in 12.2% of patients, a proportion lower than that reported in some studies ^2^^,^^16^^)^ and higher than in others ^4^^,^^10^^,^^12^. These discrepancies may be attributable to differences in the diagnostic methods used for both stroke and ASA, as well as to variations in age and comorbidity profiles across the studied populations.

The most frequent arrhythmias were atrial tachyarrhythmias, a finding similar to that reported in other studies ^4^^,^^10^^,^^15^. Several pathophysiological mechanisms have been proposed to explain this association. One hypothesis involves heterogeneous and impaired atrial conduction caused by ASA, likely due to its ability to alter the physical and electrophysiological properties of the atrial myocardium, thereby leading to interatrial and intra-atrial conduction disturbances ^17^. In addition, the presence of atypical myocytes resembling cells of the cardiac conduction system has been described in the interatrial septum, particularly at the level of the fossa ovalis. These myocytes may constitute an anatomic and electrophysiological substrate that favours the development of atrial arrhythmias ^18^. The fossa ovalis itself may also play a role in predisposing the septum to aneurysm formation ^19^. Furthermore, the mechanical motion of aneurysmal tissue secondary to pressure changes during systole and diastole has been proposed as a trigger for supraventricular arrhythmias by stretching the surrounding tissue ^10^.

The most frequent aneurysm type was type 1R, in contrast to other investigations that report type 2L as the most common ^2^^,^^12^. The reasons for this discrepancy remain unknown. When cases were grouped according to the direction of ASA deviation, a predominance of rightward deviation was observed, similar to the findings reported by Mügge *et al.*^15^. Moreover, age-stratified analysis revealed a trend toward an increasing frequency of leftward-deviating ASAs with advancing age.

Similar findings have been reported by other studies ^2^^,^^12^. To our knowledge, no prior information has been reported in the literature regarding age-related variations in the morphological characteristics of ASA.

With regard to left ventricular ejection fraction, preserved systolic function predominated in most patients, likely reflecting the high prevalence of hypertension compared with other conditions such as ischaemic heart disease and heart failure.

The detection of PFO was infrequent, probably not because of its absence, but rather due to the small size of these defects and the lack of complementary investigations specifically aimed at their detection. Only one case of ASD was identified, which exhibited a left-to-right shunt and a type 3RL ASA. It has been described that the direction of ASD flow is related to the displacement of ASA ^20^, a finding that is consistent with the observation in this case.

Mitral regurgitation was the most frequent valvular disorder, although with a lower prevalence compared with other studies ^2^^,^^4^^,^^12^^,^^13^. This may be partly explained by the higher frequency of ischaemic heart disease ^2^^,^^12^^,^^13^, dilated cardiomyopathy ^12^, and mitral valve prolapse (MVP) ^2^^,^^4^^,^^12^^,^^13^^)^ reported in those investigations, as well as by the geographic origin of the studies, with a higher prevalence of mitral regurgitation reported in North America, followed by Europe and Asia ^21^, which coincides with the regions from which those studies originated ^2^^,^^4^^,^^12^^,^^13^. Although MVP has been described as the principal mitral valvular pathology ^22^, no cases of MVP were identified in our study. Our findings may be more consistent with those of an Argentinian study, in which up to 45.5% of cases of mitral regurgitation had no identifiable aetiology, while among those with a defined cause, valvular and annular fibrosis and calcification were the most frequent, rather than MVP ^23^. 

The prevalence of aortic regurgitation was higher than that reported in other studies ^4^^,^^14^, possibly due to the older age of patients in our sample, given that its prevalence increases with advancing age ^24^.

An increased prevalence of ASA has been described among patients with MVP, suggesting a possible shared connective tissue abnormality affecting both the mitral valve leaflets and the membrane of the fossa ovalis ^7^; however, more recent studies have not confirmed this association ^14^^,^^25^. In the present investigation, two cases of aortic aneurysm were identified, similar to the findings reported by Mügge *et al.*^16^, supporting the hypothesis of an underlying connective tissue disorder as a potential cause of ASA.

Interestingly, several clinical and echocardiographic characteristics of patients with ASA differed according to the direction of aneurysmal deviation. In the group with rightward deviation, a higher frequency of atrial tachyarrhythmias was observed, along with higher values of indexed left atrial volume and E-wave velocity, consistent with a larger left atrial size. Similar findings were reported by Olivares-Reyes *et al.*^2^. In contrast, patients with leftward deviation were, on average, older, a finding also reported in another study ^2^.

The limitations of this study include its retrospective design, potential selection bias, and the fact that most patients had some form of cardiovascular disease, which limits the generalisability of the results to the general population. In addition, transoesophageal echocardiography was not performed, which may have led to an underestimation of the number of patients with ASA and PFO.

In conclusion, the prevalence of ASA was 2.0%, similar to that reported in the international literature. Female sex predominated, and hypertension was the most frequent comorbidity. Atrial tachyarrhythmias were the most common arrhythmias, type 1R aneurysm was the most frequently observed morphological subtype, and mitral regurgitation was the most prevalent valvular disorder.
